# Dataset on Women's empowerment, land and donor-driven agricultural interventions in Eastern Zambia

**DOI:** 10.1016/j.dib.2020.106113

**Published:** 2020-08-02

**Authors:** Progress H. Nyanga, Bridget Bwalya Umar, Douty Chibamba, Wilma S. Nchito

**Affiliations:** The University of Zambia, Department of Geography and Environmental Studies, Zambia

**Keywords:** Development aid modalities, Chinese aid, Norwegian aid, Conservation agriculture, Zambia

## Abstract

A survey was conducted with 235 randomly selected households to investigate women's empowerment, land and donor-driven agricultural interventions in Eastern Zambia [1] for aid programmes with (Norwegian) and without (Chinese) women empowerment goals. The survey was complemented by six focus group discussions (FGDs) and 12 key informant interviews. A triple-stream approach for focus discussions was used (i.e. women-only, men-only, and mixed gender). The results suggest that despite differing aid programme modalities, there was increased access to, and control over, productive resources by women farmers. At least 60% of the respondents reported joint ownership of all types of livestock and poultry, including large livestock such as cattle. Within households, decisions on cotton, groundnuts, and maize were made jointly by the husband and wife. Greater than 70% of the respondents in both Norwegian and Chinese aided households reported joint decision making by the husband and wife. Although both men and women farmers attended training sessions, the percentage of attending respondents was lower for Chinese-aid affiliated farmers. The majority (81% - Norwegian aid; 89% – Chinese aid) jointly earned and owned the income from cotton. When women entered into contract farming with the cotton company, operations management was performed by the entire household, and the applicable income was considered jointly earned.

Specifications Table

**Table:** 

Subject	Environmental Sciences
Specific subject area	Sustainable agriculture, landscape management and agricultural development
Type of data	Tables Direct quotations/verbatim Transcriptions
How data were acquired	Instruments: Semi-structured interview guides for the 12 key informants; household questionnaires for the random survey recipients; discussion guides for the six focus group discussions (FGDs)
Data format	Microsoft Excel (raw data)
Data collection parameters	Norwegian-funded projects were chosen to represent Western development aid in Zambia because the Norwegian Development Agency has provided more than 10 years of support for conservation agriculture, with an explicit focus on women empowerment. Chinese-funded projects were chosen to represent non-Western development aid because of their increasing influence on agricultural development in Africa (unlike Norwegian aid, the Chinese-backed programs do not focus on women empowerment). Both development aid modalities were operating in the same agricultural communities of Eastern Zambia.
Data collection description	Key informant interviews were conducted to obtain preliminary insights into the operations of three agricultural intervention case studies. The selection was made based on the researchers’ knowledge of agricultural interventions in the Eastern Province of Zambia. After the initial interviews, a survey was distributed to 235 randomly selected households. Of these, 159 participated in the Norwegian-aid intervention, and 76 participated in the Chinese-aid intervention. The questionnaire is provided as a supplementary file. The survey was complemented by six focus group discussions (FGDs) and 12 key informant interviews. A triple-stream approach (women-only, men-only, and mixed-gender) was used for the FGDs.
Data source location	Institution: The University of Zambia City/Town/Region: Lusaka Country: Zambia Physical Address: Great East Road, 32379 Main Campus
Data accessibility	Data are hosted on a public repository Repository name: Mendeley Data identification number: Direct URL to data: https://data.mendeley.com/datasets/6phshh5md4/draft?a=d96b68e8-28f8-4538-83ed-ff6c34d8e9c7.
Related research article	Umar. B. B, Nyanga P.H., Chibamba, D., Nchito W., (2020). Women's empowerment, land and donor-driven agricultural interventions in Eastern Zambia, *World Development Perspectives*, Accepted

## Data Value

• This study provides a baseline for future studies and evidence for positive social and institutional changes in women empowerment.
• The data are beneficial to Western and non-Western development aid agencies (governmental and private) and the governments receiving the aid. It also benefits development aid practitioners, local non-governmental organizations and academics.
• This data can also be used to determine factors which lead to a reduced gender gap and to determine how youths – both female and male – are influenced by the emerging changes. It can also be used to determine if gender gap reductions lead to environmental and economic sustainability and to identify trade-offs made by households when they decide to participate in different aid modalities.
• The data includes ‘voices’ from respondents involved in smallholder farming in the Eastern Province of Zambia. This area is an agriculturally important region with a large potential to contribute to the agricultural gross domestic product of the country.
• Quantitative (questionnaires) and qualitative (focus group discussions) data collection allowed for in-depth, multi-perspective analysis.

## 1 Data description

Five hundred nine respondents or observations were collected. The location and basic demographics are provided first, followed by the respondent's education status and household socioeconomic characteristics.

### 1.1 Location and basic demographics

Location and basic demographics include the serial number, district name, sex and age of the main respondents, marital status, head of household (HOH) age, spouse age, the total number of household respondents, number of male and female household members, the total number of household members < 15 years old and the total number of household members ≥ 15 years.

### 1.2 Variables for household members education status

Columns P to BA show the HOH and spouse's highest education level, English reading ability and local language reading ability. Further, the education levels of the other household members are documented (never attended school, attended/attending primary and secondary school levels, completed high school (Grade 12) or completed tertiary education). The data are separated by sex and age.

### 1.3 Institutional affiliation and land ownership

Columns BB to BE show the institutional affiliation of the household members. Norwegian-funded conservation agriculture efforts are shown in column BB [Conservation Farming Unit (CFU)] and BC [Community Markets for Conservation (COMACO)]. Affiliations to the Chinese-funded agricultural project are indicated in BD.

### 1.4 Land ownership and labour contribution by gender

The household land ownership status is provided in column BF, followed by the number of females and males that contributed to the farm labour during the 2014 - 2015 season.

### 1.5 Decision making

The intra-household decision-making data are presented in terms of the expenditure items (columns BI - BS). The expenditure categories include agriculture, food, clothes, education, fuel, medicines, remittances, groceries, grinding meal, water and other expenses. Decision-making data on the sale of crops (columns BT - CD) and livestock (columns CE - CL) are presented, followed by household decisions on income use from various sources (columns CM - CS).

### 1.6 Experimental design, materials and methods

 

### 1.7 Methods

A sequential quantitative-qualitative mixed methods design [2] was used in this study. This research is designed to provide a comprehensive understanding of the gender roles in the intra-household decision-making process by drawing on the strengths of qualitative and quantitative research methodologies.

#### 1.7.1 Data collection

During a scoping exercise in 2014, key informant interviews were conducted to obtain insights into the operations of the Conservation Agriculture Project (CAP), Community Markets for Conservation (COMACO), and China Africa Cotton Company (CACC). Afterwards, between October and November 2015, 235 randomly selected households were interviewed. Of these, 159 households participated in the Norwegian intervention projects and 76 in the Chinese projects. The survey was complemented by six focus group discussions (FGDs) and 12 key informant interviews in August 2016 using a triple-stream approach (i.e. women-only, men-only and mixed gender). The Harvard Gender Analysis Framework [3] access and control profile was used during the FGDs to ensure that the views from each gender group were vocalized without interference from the other and to enable observations of the intra- and inter-gender dynamics. Based on the survey results, key informants were interviewed about mainstreaming gender in agricultural interventions, perceptions of gender differences in technology and decision making and land inheritance patterns.

## Declaration of Competing Interest

The authors declare that they have no known competing financial interests or personal relationships which have, or could be perceived to have, influenced the work reported in this article.

## Appendix Supplementary materials

Image, application 1
