# Supplementary material for: Dataset on Women's empowerment, land and donor-driven agricultural interventions in Eastern Zambia
Source: Data Brief. 2020 Aug 2;32:106113. doi: 10.1016/j.dib.2020.106113 (PMC7424208; doi:10.1016/j.dib.2020.106113)
Supplement: Supplementary file 1 [file mmc1.pdf]

Questionnaire for Women's empowerment, land and donor-driven agricultural interventions in Eastern Zambia

Section A **Identification** (To be filled in by the enumerator at the start of the interview!!!)

1 Questionnaire code: ____ 2. District: __________________ 3. Area: _____________________
4 Village: _________________ 5. Interviewer ID: ______ 6. Date of interview__/__/2015
7 Respondent's name(s): _________________________________8_ Mobile phone: ___________________
9. Main respondent sex (M/F) ______________10. Main respondent age_____________

Section B **Basic household information**

B1 Marital status □Single □ Married □ Divorced □Widow
B2. Type of household □ Male headed □ Female headed

B3 Respondent(s) present □ Both spouses □ Male spouse only
□Female spouse(s) only □Others

B4 Age and level of Education of the Head of Household and spouse(s)

	Head of household	spouse
Age or year of birth		
Highest grade attended in school		
	1.No, 2.yes, few words,<br>3.Yes, average<br>4.Yes,most words<br>5. All words	1.No, 2.yes, few words<br>3.Yes, average<br>4.Yes, most words<br>5. All words
Ability to read in English		
Ability to read in local language		
Ability to write in English		
Ability to write in local language		

B5 What is the total number of persons in normally living in your house, including yourself?

	Male	Female	Below 15 years		15 years & above	
			Male	Female	Male	Female
Household size						
Provide the education status of your household in terms of the following:						
No. of members never gone to school						
No. of members in primary school						
No. of members in secondary school						
No. of members completed grade 12 between 2006-2015						
No. of members completed college between 2006-2015						

B6 Who owns land that your household is using? □Husband □Wife □Husband's family □Wife's family □Others (specify)
____

B7 How many household members were engaged in working on the farm the past farming season (2014/2015)? Females ___________ Males___________

Section C **Adoption and attitudes related to conservation farming/agriculture**

C1 Are you or any member of your household taking part in CFU/ conservation agriculture project activities? □Yes □No
C2 Are you or any member of your household taking part in China Africa Cotton Company/ Chipata Cotton and Oil Company) activities? □Yes □No
C3 Have you heard about conservation farming? If yes, which is your most important source of information on conservation farming?
□ Radio/Television □The Conservation Farming Unit (CFU) □Fellow Farmers
□ CASUP- Conservation Agriculture Scaling Up program of FAO and the Ministry of agriculture
□ Other sources (specify), ______________________ □Haven't heard about it

During the past 12 months: how many CFU/CACC trainings sessions did the husband and wife attend for each of the following types?			18. If you attended the training sessions, how would you assess the usefulness in terms of how much knowledge you gained?<br>1= did not learn anything<br>2= learned something that was useful and feel I/we still need some minor training<br>3= learned a lot but still feel a need for more training<br>4= learned a lot and does not need further training<br>5= don't know	
Type of CFU /CACC training session	Husband (no)	Wife (no)	Level	Reasons/explanation
Land preparation				
Input application				
Musangu				
Weeding				

C5 Can you describe briefly what Conservation Farming is? ____

____

D8 State the tillage method(s) used on the woman's plots if any in 2014/2015 season? (Multiple answers allowed)

Type of fields	Conventional hoe cultivation	Conservation farming basins	Own oxen & plough	hired or borrowed oxen and/or plough	Own oxen & Ripper	hired or borrowed oxen &/or ripper
Man's						
Woman's						
Both/Family'						

C6 Please could you estimate the area under each tillage method and the time that your household used last farming cropping season 2014/2015?

Tillage method/ Land preparations	Area (lima/ ha /acre / amount of maize seed)	Tilling the land				Weeding (1=hand only, 2= oxen weeding 3=herbicides)				
		No. of persons/ pair of oxen	No. days of tilling	Hours per days Males	Hours per day Females	Method	No. of persons/ pair of oxen	No. of days of weeding	Hours per day Females	Hours per day Males
Conventional hoe-method										
Conservation farming basins										
Own oxen &/ plough										
Borrowed/hired oxen &/or plough										
Own oxen & ripper										
Borrowed/hired oxen &/or ripper										
Other means:(specify)										

C7 What happened to the crop residues in 2014/2015 season?
 Burnt left to be grazed Left on the surface for soil improvement
 Incorporated into the soil collected for animal feed, others (specify)…………………………..

C8 Over the past 10 years what is your perception over burning of crop residues in terms of the following

	In your fields	Reason for change or lack of change	In the village generally	Reason for change or lack of change
	1=reduced<br>2=same<br>3=increased		1=reduced<br>2=same<br>3=increased	
Deliberate heaping and burning of plant residues by owners				
Burning of plant residues by owners without heaping them				
Incidences of burning of crop residues by none field owners				
Overall incidences of crop residue burning				

C9 Did you practice crop rotation in 2014/2015 season? If yes, which crops did you interchange Cotton
Cowpea Green gram Groundnuts Guar Maize Soya beans Mbambara nuts mixed beans
Sunflower Sun hemp Irish potatoes Sweet potatoes No crop rotation Others, Specify: ……………………
C10 How do you assess the fertility of your land?
 Very good Good Neither good nor poor Poor Very poor
C11 What is the tenure of your land? Customary, Titled Any other ______________________

Section D Crop production and inputs

D1. Crops and seed source

Crops	1. In the last 3 years have you grown any of the following crops? 1=yes 0=no	2. How large area did you plant to this crop?	3. Use 1=food 2=cash 3=other (specify)	4. Seed/seedling source						
				1. Own harvest Kg	2. Bartered/ exchanged Kg	3. Gift Kg	4. Bought Local market Kg	5. Bought Shop Kg	6. Bought from FISP (government) Kg	7. Other (specify) Kg
Maize										
Sorghum										
Banana										
Coffee										
Tobacco										
Cotton										
Cassava										
Sunflower										
Bullrush millet/ pearl millet										
Sesame										
Common beans										
Rice										
Green vegetables										
Others										

D2. Please estimate your crop production in the last farming season 2014/2015?

Crop	Whose crop? 1=Man 2=Woman 3=Both/family	Total Planted area (ha/ lima)	Quantity of seed (kg)	Total production (Ox-carts or 50 kg bags/20litre tin/ Meda)	Production for Sale (Ox-carts or 50 kg bags/20litre tin/ Meda)
Cassava					
Cotton					
Cowpeas					
Groundnuts					
Maize					
Soya beans					
Other beans					
Sunflower					
Sweet Potatoes					
Other crops (Specify)					

D3. Please estimate your maize production under the following tillage methods in the last farming season 2014/2015?

Tillage method	Planted Area (ha or lima or acre)			Total maize production (Ox-carts or 50 kg bags)			Production for household consumption (Ox-carts or 50 kg bags)			Production for sale (Ox-carts or 50 kg bags)		
Types of fields	Man's	Woman's	Family	Man's	Woman's	Family	Man's	Woman's	Family	Man's	Woman's	Family
Conventional hand hoe												
Conservation basins												
Oxen & ripper												
Oxen & plough												
Tractor plough												
Tractor ripper												
Other (specify)												

D4. Detailed information about maize varieties cultivated

Maize variety information						
1.Variety name						
2. Total land cultivated with this variety (acres)?						
3. Traditional/local						
4. Improved (OPV)						
5.Improved (Hybrid)						
6.If improved, was it certified?1=yes 0=no						
7. Years since fresh seeds was obtained from outside farm. 1=this season 0=Never 2-x=number of years						
8. "Creolized" seed (cross between traditional/improved)						
9. Use 1=food 2=cash						
10. Preference ranking (among different varieties) 1=best 6=least preferred						
11. Reason for growing this variety: 1=drought resistant 2=yield 3=taste 4=resistant to biotic stress 5=easily accessible 6= government subsidy						
12. Maturity 1=<90 days 2=100-120 days 3=>120 days						
13. Storability 1=Good 2=Ok 3=Not good						
14. Tastiness 1=Good 2=Ok 3=Not so tasty						
15.Input requirements 1=mineral fertilizer 2=manure fertilizer 3=pesticide 4=no requirement						
16. Drought resistant 1=very good 2=good 3=not good						
17. If bought: Price per Kg seed						
18. Are the seeds easily accessible for you? 1=yes 0=no						
19. If no: What are the main constraints? 1=price 2=not easily available 3=distance to market 4=size of seed bags for sale 5=not legally sold						
20. Quality problems?: 1=fake 2=unadapted/not suitable in area 0=no problem						

D5. When did you start planting maize under the following tillage methods in the farming season 2014/2015?

Tillage method	Date started planting			Last date of planting		
	Man's fields	Woman's field	Family fields	Man's fields	Woman's field	Family fields
CF Basins						
Conventional hand hoe						
Oxen and ripper						
Oxen and plough						

D6. Did you pay anyone to assist with any of the following tasks for any crop during the last farming season 2014/2015 ?

	No	Yes	Amount paid (expenditure in cash or kind)	Total area worked (ha)
Land tillage				
Planting				
Weeding				
Harvesting				

D7 Please specify use of inputs related to crop production in the last farming season 2014/2015 in your household?

Type of input	Quantity		
	Man's fields	Woman's field	Family fields
Fertilizer, basal (Number of 50 kg bags)			
Fertilizer, top dressing (Number of 50 kg bags)			
Manure (ox-carts)			
Herbicides (in Litres)			
Pesticides for cotton (Number of big packs)			
Other pesticides (in Litres)			
Plough bought/received			
Ripper bought/received			
Traditional hoe bought/received			
Chaka hoe bought/received			

Section E Income and expenditure

E1 Did you rent in some landduring the last farming season 2014/2015? If yes state the size of rented land and amount paid.
Size____________ (lima/ha/acre) Amount_____________ (ZMK)

E2 Did you rent out some piece of land? If yes state the size of land and amount received
Size____________ (lima/ha/acre) Amount_____________(ZMK)

E3 Please give us an estimate of the household's expenditure in a good month (highest expenditure) and bad month (lowest expenditure) during the past 12 months.

Expenditure item	Monthly expenditure (ZMK)		Who tends to decide mostlyon the budget & use of money?
	Good month	Bad month	1=Man 2=Woman 3=Both
Agriculture (seed, fertiliser, herbicides, pesticides)			
Food items (maize, oil, salt, sugar, etc)			
Clothes			
Education (fees, books, pens etc)			
Fuel, candles, matches, etc			
Medical			
Remittances given (in cash or kind)			
Glossaries (soap, lotion)			
Grinding of maize			
Water contributions			
Any other (specify)			

E4 Please give an estimate of your household's income from the following over the past 12 months

Income source		Income ZMK			Who tends to decide on the sale? (tick off)		
		Man	Woman	Both/family	Man	Woman	Both/family
Sale of Crops	Cassava						
	Cotton						
	Cowpeas						
	Groundnuts						
	Maize						
	Soya beans						
	Other beans						
	Sunflower						
	Sweet Potatoes						
	Other crops (Specify)						
Sale of animals	Cattle						
	Goats						
	Pigs						
	Chicken						
	Any Other						
Sale of animal products	Milk						
	eggs						
	Meat/+skin						
Sale of forest products (fibre, poles, honey, fire wood, wild fruits, grass, charcoal, etc)							
Other sources of income Tick off whose income)					Man	Woman	Both
Formal employment							
non-agricultural casual (piece work) work							
Agricultural casual (piece work) work							
Crafts (shoe repair, curving sewing etc) Operation period___months	Best-selling month						
	Worst selling month						
Business/Petty business Operation period___months	Best-selling month						
	Worst selling month						
Gardens Operation period ___months	Best-selling month						
	Worst selling month						
Remittances received							
Any other (specify)							

Section F Household assets

F1 Please specify the number of your house structures with of the following conditions

a. Corrugated roof____ b. Walls of burnt bricks______ c. Walls of unburnt bricks______

d. Cement floor______ e. Metal window frame(s)_______

F2 Please specify the number of livestock that is owned by the household:

Type of livestock	Quantity and ownership						
	Household leadership			Others members		Relatives elsewhere	
	Husband	Wife	Both	Man	Woman	Man	Woman
Total number of cattle							
Trained cattle							
Goats							
Sheep							
Donkeys							
Pigs							
Chickens							
Ducks							
Guinea fowl							
Pigeons							
Turkey							
Others specify)							

F3 Please specify agricultural implements in your household that are complete/currently in working condition

Type of implement	Quantity and ownership						
	Household leadership			Others household members		Relatives elsewhere	
	Husband	Wife	Both	Man	Woman	Man	Woman
Tractor							
Ox-plough Ripper							
Ridger (cuungwe)							
Cultivator (Nkolobeki)							
Oxcart							
Yoke (Joko)							
Chain							
Other tools for cultivation with draft animals (Specify)							
Traditional hoe							
Chaka hoe							
Wheelbarrow							
Other agricultural hand-tools (Specify)							

F4 Please specify any other important assets in the household.

Type of implement	Quantity and ownership						
	Household leadership			Others household members		Relatives elsewhere	
	Husband	Wife	Both	Man	Woman	Man	Woman
Bicycle							
Radio							
Television							
Mobile phones							
Generator							
Car							
Car battery							
Motor cycle							
Solar panel							
Other important assets (specify)							

Section G Food security

G1 Do you consider that your household has had enough food all the time over the past 12 months?
 yes No

G2 If no, in which months out of the past 12 months did you experience food Shortage?
Jan Feb Mar Apr May Jun Jul Aug Sep Oct Nov Dec

G3 Coping strategies in times of stress

If there have been times during the past 30 days when you did not have enough food or enough money to buy food, has your household had to:	How often have you or your household had to do this<br>0. Never<br>1. Hardly at all (<1 time/week)<br>2. Once in a while (1-2 times/week)<br>3. Pretty often (3-6 times/week)<br>4. Always (every day)	
Rely on less preferred or less expensive food?	2.1	
Borrow food, or rely on help from a relative?	2.2	
Purchase food on credit?	2.3	
Gather wild foods, "famine foods", hunt, or harvest immature	2.4	
Consume seed stock that will be needed for next season?	2.5	
Send household members to eat elsewhere?	2.6	
Send household members to beg?	2.7	
Limit portion size at mealtimes?	2.8	
Restrict consumption by adults in order for small children to eat?	2.9	
Reduce number of meals eaten in a day?	2.10	
Skip entire days without eating?	2.11	

G4 Did you receive any food aid over the past 12 months? If yes, how much in total did you receive in the household? Number of times ___Amount received each time __________Kg (Total_________)

G5 State the number of main meals that you're household has eaten over the past 24 hours?
0 1 2 3 4

G6 State the types /composition of food in the main meals that your household has eaten over the past 24 hours?

Main meals	Types/composition of foods eaten
First main meal	
Second main meal	
Third main meal	
Fourth main meal	

G7 Please list any other types of food that your household has eaten over the past 24 hours apart from the main meals listed above? 1. ____________________ 2.____________________3. ________________________
4.________________________5.______________________6.________________________

G8 Now I would like to ask you some questions about what kind of food that is consumed in this household

In the past 30 days, how often have you or your household eaten:	Frequency<br>0. Never 1. Hardly at all (<1x/week)<br>2. Once in a while (1-2x/week)<br>3. Pretty often (3-6 x/week) 4. Always (every day)	
Any food made from grains: nsima, maize, sorghum, rice, wheat, millet, bread, biscuits, or any other grain product?	84.1	
Any food made from tubers: cassava sweet potato, potatoes, carrots, or other foods made from roots or tubers?	84.2	
Any pulses (beans, lentils, peas)?	84.3	
Any vegetables? (green leafs, tomatoes, other vegetables)	84.4	
Any fruits?	84.5	
Any meat: beef, lamb, goat, wild game, fish, chicken, or other birds, liver, kidney, or other organ meats?	84.6	
Any eggs?	84.7	
Any dairy products - milk, cheese, yogurt (not including butter)?	84.8	
Any sugar or honey?	84.9	
Any oil, fat, or butter?	84.10	

G9 Has any of your household members below the age of 15 years been too ill to play or been in bed most of the time of the sickness over the past 12 months? Number ________________

Patients	Episodes	Average days of sickness
1		
2		
3		
4		

Section H Trees on the farm

H1 Please state the number of following trees that were planted in the last 12 months?

	Number of trees planted/cuttings	Source of seedlings	No. of trees that survived	No. of trees that didn't survive	Reason for death
Faidherbia Albida(musangu)					
Jathropha					
Moringa (kapulanga cisyu)					
Cassava					
Guava					

H2 Please specify the number of the following trees growing on your land

Trees	Number of trees
Mango	
Orange	
Lemon	
Guava	
Jathropha	
Moringa (kapulanga cisyu)	
Tephrosia	
Faidherbia albida (musangu tree)	
Avocado (kotapela)	
Pawpaw	
Banana	
Mulberry	

Section I Institutional aspects

I1 Did you receive any subsidized inputs (fertilizer and seed) for the cropping season 2014/2015? If yes, how much did you receive?

	Basal fertilizer		Top dressing fertilizer		Maize seed		Reasons for any gender differences.
	Male	Female	Male	Female	Male	Female	
Quantity received (kg)							
Date received							

I2 Did you receive credit (not subsidized inputs) for agricultural inputs for any crop (including cotton) in 2014/2015 past cropping season? ☐ Yes ☐ No

I3 If yes, provide information related to source of credit, nature of credit and quantity in the table below

Source	Cotton companies		Farmer Cooperative/Association		Women Club		Individuals		Others (specify)	
	Male	Female	Male	Female	Male	Female	Male	Female	Male	Female
Nature. (Cash, input, down payment)										
Quantity (kg or ZMK)										

I4 Are you or any of your household members a member of any agricultural related institution? If yes, which institution and how many members of your household are members?

Organisation	Female members	Male members
Farmer association/cooperatives and Zambia National Farmers Union (ZNFU)		
China-Africa Cotton Company (Chipata Cotton and Oil Company)		
Other cotton company		
World vision		
Woman club		
Agriculture Support Programme(ASP)		
Other _____________		

I5 Do you have a formal role in the CFU or CACC programmes? If so, what role (i.e. lead farmer, demonstration plot farmer etc.) ____

Are you under contract farming? Yes No

If yes, kindly provide the following information

	Name of the company	CACC	Other specify	
	Years of contract farming			
	Crops involved			
	Do you receive input loans 1=Yes 2=No			
	Rank your **experience** on scale of 1=strongly agree, 2=agree, 3=disagree, 4=strongly disagree 5=don't know			
	They provide me with satisfactory training and support			
	They help with the transport of the products			
	They assist in negotiating prices in the market			
	They give a higher price for the products than others			

I6 Have you been visited at your farm by a CFU extension officer during the past 12 months? If yes, how many times? ___________________________

I7 Have you been visited at your farm by a CACC extension officer the past 12 months? If yes, how many times?
